# COVID-19 and Digital Health: Evolution, Perspectives and Opportunities

**DOI:** 10.3390/ijerph19148519

**Published:** 2022-07-12

**Authors:** Marco Dettori, Paolo Castiglia

**Affiliations:** 1 Department of Medicine, Surgery and Pharmacy, University of Sassari, 07100 Sassari, Italy; 2 University Hospital of Sassari, 07100 Sassari, Italy

**Keywords:** information and communication technologies, digital health, e-health, health literacy

## Abstract

Health Communication is key to establishing an empathic relationship between health professionals and their patients. Indeed, the ability to motivate and reassure the patient often determines the success of the therapies proposed. In the past, the relationship between health professionals and patients was centred on treatment and assistance, and health information came in the form of education campaigns based on signage (large posters, etc.). Subsequently, social and cultural changes gave rise to new ways of communicating science: from signage to magazines and television programmes devoted to health issues, through the use of social networks. In particular, fast and cheap access to the Internet and the vast number of app users have made the Web an effective communication tool. Given the potential of technology in the information-seeking process, the use of online channels by health institutions is a valuable tool for divulging medical and scientific knowledge. As a consequence, prompted by the need to provide fast and reliable information to the population, public institutions have adopted such innovative methods as the use of Information and Communication Technologies (ICTs) to convey health-related content. This practice, known as digital health or e-health, provides healthcare information using digital tools (e.g., Websites and social networks), delivered in an easy-to-understand language in order to reach various population groups and bring about better health conditions for all, hence the importance of acquiring and reinforcing communication skills in the healthcare field, where correct and effective communication immediately translates into a benefit for the professionals themselves and for their patients.

## Background

The need to establish an effective interaction with users means that Health Communication is one of the basic principles of healthcare. Indeed, the trust between the patient and the healthcare provider is a prerequisite for the preventive and therapeutic process both in terms of information and health education [1,2].

In the past, health communication was regarded as a mere means of transferring information from an issuer, regarded as educator, to a receiving audience who was subordinate to the former [1,3]. Under this premise, the message conveyed by the health professional assumed that the socially accepted relevance of the health content was capable of modifying the individual’s behaviour.

In the twentieth century, healthcare information was primarily communicated through signage (posters, etc.) and focused on hygiene regulations concerning the prevention of deadly diseases such as polio, smallpox and cholera, or on more specific topics such as the promotion of safety at work [3,4,5]. Subsequently, the social and cultural transformations over the decades solicited a renewed approach to health issues, modifying the contents and models of health disclosure. In fact, there has been a shift from information of an institutional nature aimed indiscriminately at the general public to a model in which health-related communication sources are accessible to population groups based on their varying degrees of interest [5,6]. As a result, the interaction between health professionals and users has shifted from a user-subordinate relationship to a complementary one. Thus, the unidirectionality of health-related messages has been replaced by the bidirectionality of information no longer passively acquired by users but autonomously sought out. In this context, the digital revolution brought about by Information and Communication Technologies (ICTs) has sanctioned a change in the interactions between people [7,8].

In this era, characterised by the advent of social networks as new forms of communication and public participation, the average user has become more familiar with IT tools, making web interaction more participatory.

The current COVID-19 pandemic has further contributed to the increase in the use of digital platforms both for the response to the need for interaction and for needs related to smart working and distance learning. However, the percentage increase in the online audience could also be explained by the fact that, nowadays, a significant number of people use the web to investigate or comment on a variety of topics with particular reference to health issues [9,10,11].

This phenomenon, described by the World Health Organization as the Infodemic, is a response to an identity need according to which the individual safeguards his state of health through independent access to ICTs and to sources dealing with well-being and healthy lifestyles. This exposes the user to the vast amount of information and content available online [12,13,14,15,16]. Indeed, while current digital communication systems have led to an increase in peer-to-peer interaction, they have also facilitated the proliferation of information that is often distorted and capable of confusing the general public.

Moreover, the exponential increase in information available online (including health-related content) and the ease of contributing personal opinions and considerations, which are sometimes misleading and lacking scientific evidence, has led to a delegitimisation of the authoritativeness of health information divulged by health professionals [7,9,16,17].

Social media and information technologies have led to a process whereby all opinions and content available online appears to have the same value. This is of concern when one considers that so many health-related topics are currently being discussed on the Web, especially vaccine-related issues, which have been of increasing interest in recent years [7,14,16]. In this regard, the presence of anti-vaccination content on the Web has contributed to a wide and rapid spread of hearsay, false myths and inaccurate information, resulting in an increased perception of vaccine risk and a consequent low adherence to immunisation programmes. According to the ‘outrage’ theory coined by the American sociologist Peter Sandman, a fundamental role in risk perception is played by the emotional component surrounding the event [18]. In this sense, risk perception not only has an individual component characteristic of the individual subject but also a socio-cultural component directly connected to the context and the social group to which one belongs [19,20,21]. This phenomenon is therefore amplified by the online community, where the presence of health-related information spread by multiple sources generates a ‘common feeling’ that superimposes itself on the individual’s personal processing and influences his or her health decisions [22,23,24,25,26].

As a result of the aforementioned, the expansion of the mass media, together with the evolution of the Web, has necessitated an improvement in the means available to Public Health, which has had to turn to ICT as a strategy to divulge evidence-based medical-scientific knowledge. In fact, thanks to the potential offered by the Internet in the process of seeking health information, the use of online channels by health institutions may guarantee the spread of medical-scientific knowledge to and among user-patients. Known as digital health or e-health, this practice delivers healthcare through the use of digital tools (e.g., websites and social networks) in an easy-to-understand language with the aim of (i) boosting the spread of high-quality health information; (ii) involving citizens/patients, enabling them to become as responsible as possible for their own health; and (iii) raising awareness on the importance of preventive strategies.

One example of health communication is embodied by the Italian network VaccinarSì, which, by publishing regional websites, informs the public about the main vaccine-preventable infectious diseases, vaccinations available, immunisation programmes and vaccination calendars, while continuously carrying out training and counselling activities for health workers and citizens. [7,9,14].

Of particular importance in this context is the relationship between health literacy and the proper management of one’s own health. This concept, developed in the early 1970s in the English-speaking world, has been expanded upon by numerous scholars, leading to the present definition of health literacy as the set of cognitive and social skills that motivate individuals by enabling them to access, understand and use information in order to support and safeguard their own health [27,28,29]. According to the definition given by the World Health Organization, health literacy implies the attainment of a level of knowledge, individual skills and self-confidence that motivates individuals to take action to improve their own and the community’s health, changing their personal lifestyle and living conditions. As such, it is deemed the best empowerment strategy to improve an individual’s ability to access and use information effectively [24].

However, for topics such as those concerning the management of one’s own health, the mere recognition of user independence to autonomously retrieve health-related information is insufficient to induce correct practices. This is shown by the ever-frequent reluctance to adhere to vaccination programmes. In fact, while the role of vaccination as a powerful life-saving tool in the fight against infectious diseases has been widely highlighted in the fight against COVID-19, there is still a discrepancy between the scientific evidence on the efficacy of vaccines and the public’s perception of their risk [29]. This suggests the need to devise and implement health policies and interventions adapted to different levels of Health Literacy in order to enable the most vulnerable population groups to better cope with health challenges without the support of health professionals.

Thus, in order to communicate science, the use of a traditional approach, based on empathy, listening and rapport, together with the opportunities offered by ICT as a multiplier of correct health information, is the best combination for attaining rational awareness and informed participation.

## Data Availability

Not applicable.

## References

[1] Weiner J.P. (2012). Doctor-patient communication in the e-health era. Isr. J. Health Policy Res..

[2] Llorente C., Revuelta G., Carrió M., Porta M. (2019). Scientists’ opinions and attitudes towards citizens’ understanding of science and their role in public engagement activities. PLoS ONE.

[3] Salmon C.T., Poorisat T. (2020). The Rise and Development of Public Health Communication. Health Commun..

[4] Stefanelli P., Bellino S., Fiore S., Fontana S., Amato C., Buttinelli G., Regional Reference Centres of the National Surveillance System for Acute flaccid paralysis (2019). Hospital dis-charges-based search of acute flaccid paralysis cases 2007–2016 in Italy and comparison with the National Surveillance System for monitoring the risk of polio reintroduction. BMC Public Health.

[5] Bravo P., Edwards A., Barr P.J., Scholl I., Elwyn G., McAllister M. (2015). Conceptualising patient empowerment: A mixed methods study. BMC Health Serv. Res..

[6] Arghittu A., Dettori M., Masia M.D., Azara A., Dempsey E., Castiglia P. (2019). Social deprivation indexes and anti-influenza vaccination coverage in the elderly in Sardinia, Italy, with a focus on the Sassari municipality. J. Prev. Med. Hyg..

[7] Arghittu A., Dettori M., Dempsey E., Deiana G., Angelini C., Bechini A., Bertoni C., Boccalini S., Bonanni P., Cinquetti S. (2021). Health Communication in COVID-19 Era: Experiences from the Italian VaccinarSì Network Websites. Int. J. Environ. Res. Public Health.

[8] Kapoor K.K., Tamilmani K., Rana N.P., Patil P.P., Dwivedi Y.K., Nerur S. (2018). Advances in Social Media Research: Past, Present and Future. Inf. Syst. Front..

[9] Castiglia P., Arghittu A. (2022). New Insight in Vaccination and Public Health: A Commentary from Special Issue Editors. Vaccines.

[10] Marina Ocaña J., Feliz Murias T., Marina Ocaña J., Feliz Murias T. (2018). Percepciones en la búsqueda de información y educación para la salud en entornos virtuales en español. Rev. Esp. Salud Publica.

[11] Parmeshwar N., Reid C.M., Park A.J., Brandel M.G., Dobke M.K., Gosman A.A. (2018). Evaluation of Information Sources in Plastic Surgery Decision-making. Cureus.

[12] Stahl J.-P., Cohen R., Denis F., Gaudelus J., Martinot A., Lery T., Lepetit H. (2016). The impact of the web and social networks on vaccination. New challenges and opportunities offered to fight against vaccine hesitancy. Méd. Mal. Infect..

[13] Bonito B., Balzi D., Boccalini S., Bonanni P., Mereu G., Santini M.G., Bechini A. (2021). Descriptive Observational Study of Tdap Vaccination Adhesion in Pregnant Women in the Florentine Area (Tuscany, Italy) in 2019 and 2020. Vaccines.

[14] Arghittu A., Deiana G., Dettori M., Dempsey E., Masia M.D., Plamieri A., Spano A.L., Azara A., Castiglia P. (2021). Web-based analysis on the role of Digital Media in Health Communication: The experience of VaccinarSinSardegna Website. Acta Biomed..

[15] Betsch C., Brewer N.T., Brocard P., Davies P., Gaissmaier W., Haase N., Leask J., Renkewitz F., Renner B., Reyna V.F. (2012). Opportunities and challenges of Web 2.0 for vaccination decisions. Vaccine.

[16] Dettori M., Arru B., Azara A., Piana A., Mariotti G., Camerada M.V., Stefanelli P., Rezza G., Castiglia P. (2018). In the Digital Era, Is Community Outrage a Feasible Proxy Indicator of Emotional Epidemiology? The Case of Meningococcal Disease in Sardinia, Italy. Int. J. Environ. Res. Public Health.

[17] Castiglia P., Dettori M. (2022). Second Edition of Special Issue “Strategies and Evidence in Health Communication: Evidence and Perspectives”. Int. J. Environ. Res. Public Health.

[18] Sandman P. (2012). Responding to Community Outrage: Strategies for Effective Risk Communication.

[19] Arghittu A., Dettori M., Azara A., Gentili D., Serra A., Contu B., Castiglia P. (2020). Flu Vaccination Attitudes, Behaviours, and Knowledge among Health Workers. Int. J. Environ. Res. Public Health.

[20] World Health Organization Managing the COVID-19 Infodemic: Promoting Healthy Behaviours and Mitigating the Harm from Misinformation and Disinformation. https://www.who.int/news/item/23-09-2020-managing-the-covid-19-infodemic-promoting-healthy-behaviours-and-mitigating-the-harm-from-misinformation-and-disinformation.

[21] Dettori M., Arghittu A., Deiana G., Azara A., Masia M.D., Palmieri A., Spano A.L., Serra A., Castiglia P. (2021). Influenza Vaccination Strategies in Healthcare Workers: A Cohort Study (2018–2021) in an Italian University Hospital. Vaccines.

[22] Costantino C., Casuccio A., Restivo V. (2020). Vaccination and Vaccine Effectiveness: A Commentary of Special Issue Editors. Vaccines.

[23] Adams K., Corrigan J.M. (2003). Institute of Medicine. Priority Areas for National Action: Transforming Health Care Quality. Committee on Identifying Priority Areas for Quality Improvement.

[24] Nielsen-Bohlman L., Panzer A.M., Kindig D.A., Institute of Medicine (US) Committee on Health Literacy (2004). Health Literacy: A Prescription to End Confusion.

[25] Dettori M., Pittaluga P., Busonera G., Gugliotta C., Azara A., Piana A., Arghittu A., Castiglia P. (2020). Environmental Risks Perception Among Citizens Living Near Industrial Plants: A Cross-Sectional Study. Int. J. Environ. Res. Public Health.

[26] Masia M.D., Dettori M., Deriu G.M., Soddu S., Deriu M., Arghittu A., Azara A., Castiglia P. (2021). Microbial Monitoring as a Tool for Preventing Infectious Risk in the Operating Room: Results of 10 Years of Activity. Atmosphere.

[27] Smith B., Magnani J.W. (2019). New technologies, new disparities: The intersection of electronic health and digital health literacy. Int. J. Cardiol..

[28] World Health Organization. Regional Officer for Europe Health Literacy—The Solid Fact. https://apps.who.int/iris/bitstream/handle/10665/128703/e96854.pdf.

[29] Dettori M., Arghittu A., Castiglia P. (2022). Knowledge and Behaviours towards Immunisation Programmes: Vaccine Hesitancy during the COVID-19 Pandemic Era. Int. J. Environ. Res. Public Health.

